# Early-Life Gut Bacteria Associate with IL-4−, IL-10− and IFN-γ Production at Two Years of Age

**DOI:** 10.1371/journal.pone.0049315

**Published:** 2012-11-20

**Authors:** Maria A. Johansson, Shanie Saghafian-Hedengren, Yeneneh Haileselassie, Stefan Roos, Marita Troye-Blomberg, Caroline Nilsson, Eva Sverremark-Ekström

**Affiliations:** 1 Department of Immunology, The Wenner-Gren Institute, Arrhenius Laboratories for Natural Sciences, Stockholm University, Stockholm, Sweden; 2 Department of Microbiology, Uppsala BioCenter, Swedish University of Agricultural Sciences, Uppsala, Sweden; 3 Department of Clinical Science and Education, Södersjukhuset, Karolinska Institutet and Sachs' Children's Hospital, Stockholm, Sweden; Instutite of Agrochemistry and Food Technology, Spain

## Abstract

Microbial exposure early in life influences immune maturation and potentially also the development of immune-mediated disease. Here we studied early-life gut colonization in relation to cytokine responses at two years of age. Fecal samples were collected from infants during the first two months of life. DNA was extracted from the fecal samples and *Bifidobacterium (B.) adolescentis*, *B. breve*, *B. bifidum*, a group of lactobacilli (*L. casei*, *L. paracasei* and *L. rhamnosus*) as well as *Staphylococcus (S.) aureus* were detected with real time PCR. Peripheral mononuclear cells were stimulated with phytohaemagglutinin (PHA) and numbers of IL-4−, IL-10− and IFN-γ secreting cells were evaluated using ELISpot. We further stimulated peripheral blood mononuclear cells with bacterial supernatants *in vitro* and assessed the IL-4−, IL-10− and IFN-γ inducing capacity by flow cytometry and ELISA. Early *S. aureus* colonization associated with higher numbers of IL-4− (p = 0.022) and IL-10 (p = 0.016) producing cells at two years of age. In contrast to colonization with *S. aureus* alone, co-colonization with lactobacilli associated with suppression of IL-4− (p = 0.004), IL-10− (p = 0.004) and IFN-γ (p = 0.034) secreting cells. *In vitro* stimulations of mononuclear cells with bacterial supernatants supported a suppressive role of *L. rhamnosus* GG on *S. aureus*-induced cytokine responses. We demonstrate that the early gut colonization pattern associates with the PHA-induced cytokine profile at two years of age and our *in vitro* findings support that specific bacterial species influence the T helper cell subsets. This suggests that dysbiosis in the early microbiota may modulate the risk of developing inflammatory conditions like allergy.

## Introduction

The gut microbiota performs necessary metabolic functions such as production of short chain fatty acids and synthesis of vitamins. It also influences the maturation of the immune system after birth, which is clearly illustrated in studies of germ-free (GF) animals [1]. GF mice have fewer intestinal dendritic cells (DC) [2] and mice with a restricted microbiota have less plasmacytoid DCs [3]. Moreover, while segmented filamentous bacteria induce IL-17 and IL-22 producing CD4^+^ cells in the lamina propria [4], the immunomodulatory polysaccharide A, produced by *Bacteroides fragilis,* induces Foxp3^+^ IL-10-producing T regulatory cells [5]. Lathrop et al. recently demonstrated that the peripheral T cell population, besides the thymic self/nonself discrimination instructions, further is educated by the colonic microbiota [6].

Recently, the microbiota has also been shown to influence immune responses to infections as well as the development of non-infectious conditions. The response towards respiratory tract influenza is altered in antibiotic treated animals suggesting the importance of the microbiota in directing the immune responses at other sites than the gut [7]. In addition, the microbiota also seems to influence development of autoimmune disease [8] and inflammatory bowel disease (IBD) [9] in mice.

Much less is known about how the microbiota influences the human immune system. Although a failure in tolerating the intestinal bacteria is suggested in the pathogenesis of IBD [10], and an altered early-life colonization pattern associates with the development of allergic diseases [11]–[14], the underlying mechanisms of microbiota-mediated immune modulation in humans need to be further investigated. Early colonization with bifidobacteria has been associated with increased secretory IgA in saliva [15] whereas lactobacilli and bifidobacteria colonization associates with lower cytokine responses and increased Foxp3 expression following *in vitro* allergen stimulation [16]. Early *Bacteroides fragilis* colonization seems to associate with immune function also in humans. Infants colonized with *Bacteroides fragilis* early in life had more IgA-producing cells in infancy [17], spontaneous IFN-γ production and reduced pro-inflammatory responses following LPS stimulation early in life compared to non-colonized infants [15]. In addition, stimulating human immune cells *in vitro* with bacterial species have demonstrated species-specific immunostimulatory capacities [18]–[20].

We have previously reported that infants colonized with lactobacilli (*Lactobacillus (L.) rhamnosus, L. paracasei, L. casei*) and *Bifidobacterium (B.) bifidum* early in life were significantly less often allergic at five years of age, whereas the opposite tendency was seen for *Staphylococcus (S.) aureus* colonization [14]. Therefore, we wanted to investigate if early-life colonization with these species of bacteria, influences immune responses during childhood. Due to the association between the gut microbiota and T cell development/maturation we choose to stimulate peripheral blood mononuclear cells (PBMC) with the general T cell stimuli phytohaemagglutinin (PHA) and assessed IFN-γ and IL-4 as these cytokines are signature cytokines favoring cell mediated and humoral immunity, respectively, whereas IL-10 was investigated due to its potentially regulatory function. Further, we performed *in vitro* stimulations of peripheral-blood mononuclear cells (PMBCs) with bacterial supernatants to investigate how these species directly induce IL-4−, IL-10− and IFN-γ production in CD4+ T cells.

## Materials and Methods

### Subjects and isolation of blood PBMCs

For this study, a total of thirty children were included from a larger prospective study cohort [21]–[22]. Here, children were included based on availability of fecal samples at several occasions during the first two months of life as well as availability of mononuclear cells from two years of age. All infants were healthy, born term (median weeks 40, range 38–43) and had normal birth weights (mean 3,6 kg, range 2,7–4,8). Thirteen (n = 13) of the children had allergic parents and seventeen (n = 17) had non-allergic parents. The study was approved by the Human Ethics Committee at Huddinge University Hospital, Stockholm (Dnr 75/97, 331/02), and the parents provided informed verbal consent. No written documentation of the participants informed approval was required, which was agreed to by the Human Ethics Committee and was according to the regulations at the time of the initiation of the study. The midwife at the maternity ward asked families expecting a child if they were interested in participating in the study. If so, the pediatrician (C.N.) in charge contacted them, gave further information and invited them to a seminar on allergies. If still interested to participate, appointments were made for blood sampling of the parents, when approval of their participation was documented.

Mononuclear cells from venous blood sampled at two years of age, were separated within 24 hrs after collection, by Ficoll-Paque (Pharmacia-Upjohn, Uppsala, Sweden) gradient centrifugation. The cells were resuspended in tissue culture medium (RPMI 1640 Hepes containing 10% heat inactivated fetal calf serum and 25 µg/mL of gentamycin and 2 mm L-glutamine), supplemented with 10% dimethyl sulphoxide (DMSO), frozen and stored in liquid nitrogen.

PBMCs from healthy adult blood donors were obtained and processed as above. All donors gave their written informed consent to participate, which was approved of by the Regional Ethics Committee in Stockholm (Dnr 04-106/1).

### ELISpot for quantification of cytokine secreting cells after stimulation of PBMCs

Briefly, nitrocellulose plates (Millipore Corp., Bedford, MA, USA) were coated and incubated over night with anti-human monoclonal antibodies (mAbs) to IL-4, IL-10 and IFN-γ (Mabtech, Nacka, Sweden), at a concentration of 10 µg/ml, as described in detail elsewhere [23]. Cells were thawed and washed prior to being cultured in triplicate wells at a concentration of 10^6^ cells/ml with or without phytohaemagglutinin (PHA, 1 µg/ml, Murex Diagnostics Ltd, Dartford UK) for 4 hrs in round-bottomed plates, before being transferred to coated ELISpot plates and incubated for 42 hrs. Subsequently, the cells were washed away and biotinylated mAbs (IL-4, IL-10 and IFN-γ (Mabtech, Nacka, Sweden) were added and incubated for 2 hrs at room temperature (RT). Thereafter, color-developing buffer was added to allow development of spots following incubation at RT. After drying of the plates, counting of spots was performed using computerized ELISpot counter (Autoimmun Diagnostica GmbH, Strassberg, Germany, and software AID). The number of cytokine secreting cells in medium control was subtracted from the number of cytokine producing cells following PHA-stimulation and was expressed as cells per 10^5^ cells.

### Real time PCR for detection of bacteria in fecal samples

The methods for extraction of DNA from fecal samples and detection of bacterial species have previously been published in detail [12]. Infant fecal samples, collected at 1 and 2 weeks as well as 1 and 2 months of age, were brought to the hospital on ice and were stored at −70°C. DNA from the fecal samples was extracted using the Qiamp DNA Stool Mini Kit™ protocol increasing the bacterial DNA of the human DNA (Qiagen, Hilden, Germany). Measurement of extracted nucleic acid concentration was performed with Bio-Rad Smartspec (Bio-Rad Laboratories, Hercules, CA, USA) at 260 nm using Bio Rad trUView Disposable Cuvettes (Bio-Rad Laboratories).

Analyses of bacterial species were performed with Real time PCR, using SYBR Green chemistry with primer pair sequences and concentrations previously published [12]. Primer pairs used targeted, *B. adolescentis*, *B. bifidum*, *B. breve*, a group of lactobacilli (*L. casei*, *L. paracasei*, *L. rhamnosus*) [12] and *S. aureus* [24]. *L. casei*, *L. paracasei*, *L. rhamnosus* was detected with one primer pair and are referred to as “lactobacilli” from now on. As standards and positive control, reference bacterial DNA was used.

Real time PCR, for bacterial detection and amounts, was performed in 96 well detection plates in ABI prism 7000 using the Absolute Quantification protocol in 7000 SYSTEM software version 1.2.3f2 (Applied Biosystems, Stockholm, Sweden) together with standard curves ranging from 5 ng to 50 fg. Samples were analyzed in triplicates and mean C_T_ values above 35 were considered negative to avoid detection of false positives.

### Generation of bacterial supernatants

*L. rhamnosus* and *S. aureus* were used for the stimulation experiments: *L. rhamnosus* GG (ATCC 53103; isolated from the probiotic product Culturelle), and *S. aureus* 161.2 (producing Staphylococcal enterotoxin A and H). *S. aureus* strain is a kind gift from Åsa Rosengren, The National Food Agency, Uppsala, Sweden, who also has screened the strain for toxin genes by using PCR. The lactobacilli were cultured in MRS broth (Oxoid) at 37°C for 20 h and the staphylococci in BHI broth (Merck) at 37°C for 72 h still culture. The bacteria were pelleted by centrifugation at 14 000 g where after the supernatants were sterile filtered (0,2 µm) and frozen at −20°C until used.

### In vitro stimulation of PBMCs with bacterial supernatants

PBMCs from healthy adult blood donors were thawed and washed prior to being cultured at a concentration of 10^6^ cells/ml with 2,5% of bacterial supernatants from either *S. aureus* 161.2 and/or *L rhamnosus GG,* or left in culture medium for 6 h before addition of protein-transport inhibitor monensin (GolgiStop, BD Biosciences) over night for intracellular detection of cytokines. Supernatants from the same cultures were collected and stored at −85°C until cytokine analyses.

### FACS analysis of cytokine-producing T helper cells

A panel of FITC-, PE-, PerCP- and APC-conjugated mAbs for staining of CD4, IL-4, and IFN-γ were used, all from BD Biosciences. Cells were harvested and stained according to standard procedures for surface antigens. For intracellular cytokine detection, cells were fixed and permeabilized prior to staining. Gating was performed on the basis of forward and side scatter properties for live cells followed by specific gating of CD4^+^-cell cytokine production within the lymphocyte gate. Data were analyzed with FlowJo where frequencies of cytokine-producing T helper cells were determined after reduction of background percentages in cultures in medium alone. Within the live gate, cell viability was evaluated by 7AAD-binding (BD Via-Probe™) and did not differ significantly among donors and between stimulations.

### IL-4, IL-10 and IFN-γ ELISA

Determination of soluble IL-4, IL-10 and IFN-γ was made by using ELISA kits (all reagents from Mabtech, Sweden except recombinant IFN-γ which was obtained from National Biological Standards Board), according to the manufacturer's instructions. The limit of detection was 8 pg/ml for IL-4, 3 pg/ml for IL-10 and 20 pg/ml for IFN-γ.

### Statistics

To assess differences in cytokine secreting cells, among colonized and non-colonized infants, Mann Whitney U-test was used. Further, to investigate correlations between cytokine secreting cells and the relative amounts of the bacterial species, Spearman Rank Correlation test was employed. Additionally, Kruskal-Wallis ANOVA was used to investigate differences cytokine numbers with regards to co-colonization with the different species investigated and also for the bacterial supernatant stimulated PBMCs. If significant, Mann Whitney U-test was used to investigate which groups differed. Only results where trends and differences were consistent over time are reported.

## Results

### Lactobacilli-, bifidobacteria- and S. aureus colonization during the first months of life

Infant colonization during the first two months of life, was determined using RT-PCR with specific primer pairs and is shown in Table 1. Out of the species investigated, the *Lactobacillus* group consisting of *L. casei*, *L. paracasei*, and *L. rhamnosus*, was the least detected one, found only in 21,4% of the fecal samples and *S. aureus* was the most frequently detected species found in 53,6% of the samples the first week of life. The lactobacilli frequency increased during the first two months of age and was found in 50% of the fecal samples at two-month olds, as did *S.aureus* frequency, which increased, to approximately 70%.

**Table 1:** Percentages of infants colonized with respective bacteria.

	Week 1.		Week 2.		Month 1.		Month 2.	
		%		%		%		%
***B. adolescentis***	11/28	39,3	9/27	33,3	8/24	33,3	9/26	34,6
***B. bifidum***	8/28	28,6	10/27	37,0	9/24	37,5	14/26	53,8
***B. breve***	8/28	28,6	7/27	25,9	8/24	33,3	8/26	30,7
**Lactobacilli**	6/28	21,4	9/27	33,3	8/24	33,3	13/26	50
***S. aureus***	15/28	53,6	19/27	70,4	18/24	75	19/26	73,1

Of the bifidobacteria investigated, *B. adolescentis* was the most common at one week of age, detected in 39,3% of the infants, whereas *B. bifidum* was the most frequently detected bacterium at two months of age. The bifidobacteria frequencies remained stable throughout the two months, except *B. bifidum* frequency, which increased to 53,4% at the age of two months. *B. breve* was detected in approximately 25–30% of the infants throughout the first two months after birth.

### Early colonization with lactobacilli associates with fewer cytokine- secreting cells at two years of age

**Figure 1:**
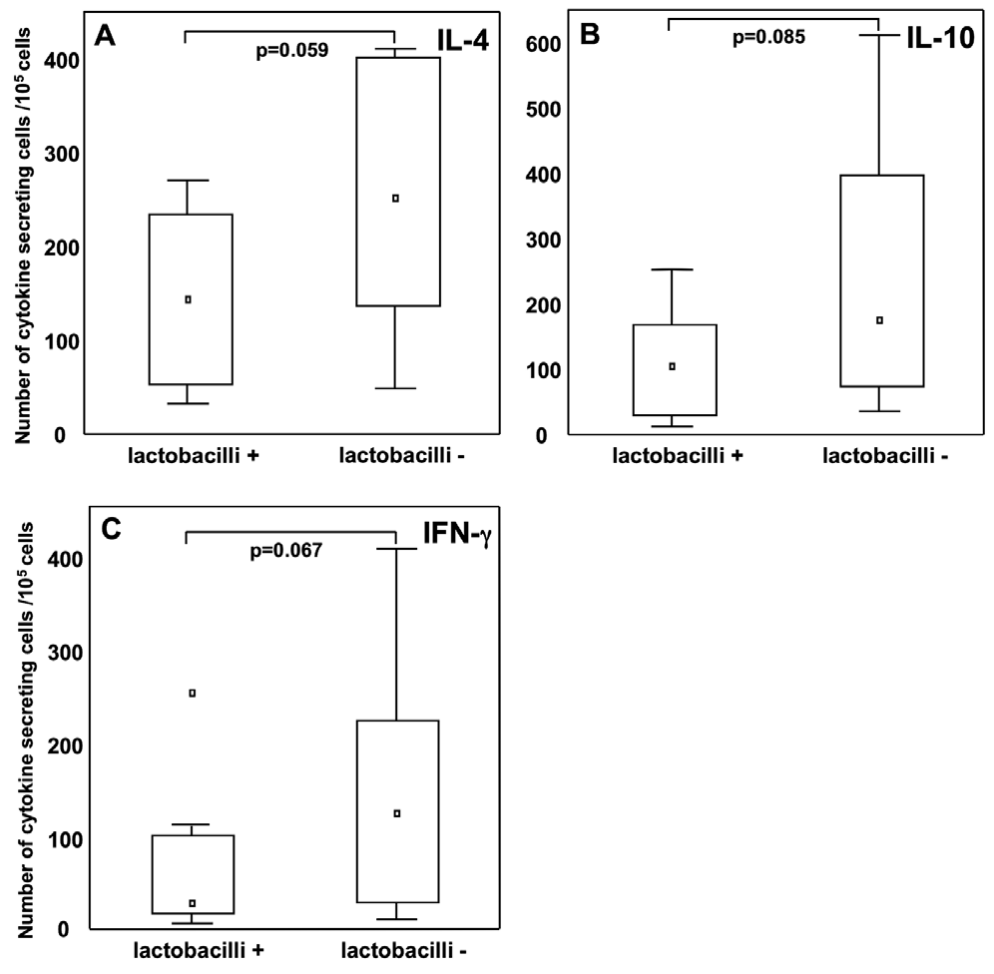
Lactobacilli colonization at 2 weeks of age in relation to cytokine secreting cells, after *in vitro* PHA stimulation at age two. Infants with (n = 9) or without (n = 18) lactobacilli at 2 weeks of age in relation to (A) IL-4−, (B) IL-10− and (C) IFN-γ producing cells after PHA stimulation. Boxes cover 25^th^ to 75^th^ percentile and the central square being the median value. Whiskers extend to non-outlier maximum and minimum and squares represents outliers.

As we have observed that lactobacilli are more frequently detected in the fecal microbiota of children remaining non-allergic later in life [12], [14], we wanted to study how the early-life colonization pattern associates with immune responses in childhood. We found that early-life colonization with *L. casei*, *L. paracasei*, and *L. rhamnosus*, referred to as lactobacilli, was inversely associated with cytokine-secreting cell numbers following *in vitro* PHA stimulation. Infants colonized with lactobacilli at two weeks of age tended to have fewer IL-4− (p = 0.059), IL-10−(p = 0.085) and IFN-γ (p = 0.067) producing cells at two years of age (Fig. 1A–C). Similarly, lactobacilli colonization at one month tended to associate with lower numbers of IL-4 (p = 0.065) and IL-10 (p = 0.075), whereas lactobacilli colonization at the other time points investigated did not associate with lower numbers of cytokine-producing cells.

Moreover, the relative amounts of lactobacilli at two weeks (p = 0.042), one month (p = 0.040) and two months (p = 0.049) correlated with lower numbers of IL-4 producing cells at age two (Table 2).

**Table 2:** Relative amounts of infant gut bacterial species in relation to cytokine secreting cells at two years of age.

	*Lactobacilli*		*B. bifidum*		*B. breve*		*B. adolescentis*		*S. aureus*	
	Spearman R	*P*-value	Spearman R	*P*-value	Spearman R	*P* -value	Spearman R	*P* -value	Spearman R	*P* -value
**Week 1**										
**IL-4**	−0.126	0.522	−0.334	0.083	−0.202	0.302	−0.001	0.995	0.064	0.746
**IL-10**	0.025	0.897	−0.238	0.223	−0.325	0.091	−0.099	0.617	0.257	0.186
**IFN-γ**	−0.186	0.433	−0.141	0.474	−0.038	0.850	−0.019	0.923	0.143	0.469
**Week 2**										
**IL-4**	**−0.394**	**0.042**	−0.353	0.071	−0.269	0.175	−0.016	0.935	0.332	0.091
**IL-10**	−0.375	0.054	−0.299	0.130	−0.364	0.062	0.014	0.944	**0.462**	**0.015**
**IFN-γ**	−0.331	0.091	−0.100	0.619	0.086	0.664	0.167	0.406	0.119	0.553
**Month 1**										
**IL-4**	**−0.423**	**0.040**	−0.310	0.141	−0.226	0.288	0.059	0.784	0.181	0.396
**IL-10**	−0.393	0.057	−0.249	0.241	−0.325	0.122	0.149	0.488	0.323	0.124
**IFN-γ**	−0.151	0.482	−0.298	0.157	0.134	0.531	0.269	0.203	0.210	0.324
**Month 2**										
**IL-4**	**−0.390**	**0.049**	−0.243	0.232	−0.301	0.136	−0.066	0.750	0.334	0.096
**IL-10**	−0.305	0.130	−0.357	0.074	−0.351	0.079	−0.072	0.722	0.381	0.055
**IFN-γ**	0.153	0.455	0.011	0.958	0.070	0.732	0.224	0.271	0.118	0.566

Week 1 n = 28, Week 2 n = 27, Month 1 n = 24, Month 2 n = 26.

Significant *P*-values are shown in bold text.

### Colonization with the bifidobacteria investigated did not associate with cytokine secreting cell numbers

In addition to colonization with lactobacilli, bifidobacteria have been associated with less allergy development [11] and are common colonizers of the newborn gut. However, neither colonization with *B. adolescentis, B. bifidum* and *B. breve* nor their relative amounts associated with cytokine-producing cell numbers at age two (not shown and Table 2).

### Early S. aureus colonization associates with increased numbers of cytokine secreting cells at age two

**Figure 2:**
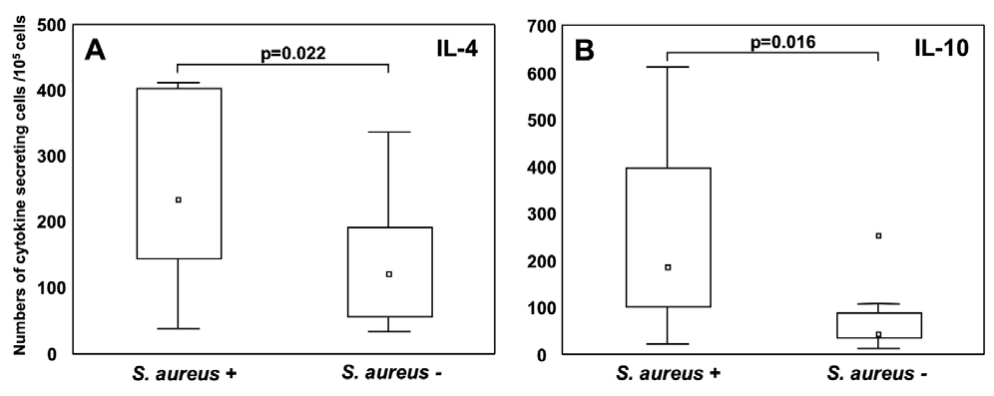
*S. aureus* colonization at 2 weeks of age in relation to cytokine secreting cells, after *in vitro* PHA stimulation at age two. Infants with (n = 19) or without (n = 8) *S. aureus* at 2 weeks of age in relation to (A) IL-4− and (B) IL-10 producing cells after PHA stimulation. Boxes cover 25^th^ to 75^th^ percentile and the central square being the median value. Whiskers extend to non-outlier maximum and minimum, squares and stars represents outliers and extremes, respectively.

In contrast to the lactic acid bacteria, *S. aureus* colonization has been associated with development of various allergic manifestations [25]. Compared to the lactobacilli, early *S. aureus* colonization was associated with a reverse pattern of cytokine producing cells at age two after PHA stimulation. *S. aureus* colonization at two weeks of age associated with significantly increased numbers of IL-4− (p = 0.022) and IL-10 (p = 0.016) producing cells (Fig. 2A–B). Similarly, colonization with *S. aureus* at one- (p = 0.047) and two months (p = 0.035) of age associated with significantly increased numbers of IL-10 secreting cells (not shown) whereas a tendency for increased numbers of IL-4 secreting cells was present among the infants colonized with *S. aureus* at two months of age (p = 0.09) (not shown). No associations between *S. aureus* colonization and IFN-γ producing cell numbers were observed.

Moreover, the relative amounts of *S. aureus* weakly associated with increased numbers of IL-10 secreting cells at two years of age (Table 2).

### Co-colonization with lactobacilli dampens the S. aureus associated cytokine producing cell numbers at two years of age

**Figure 3:**
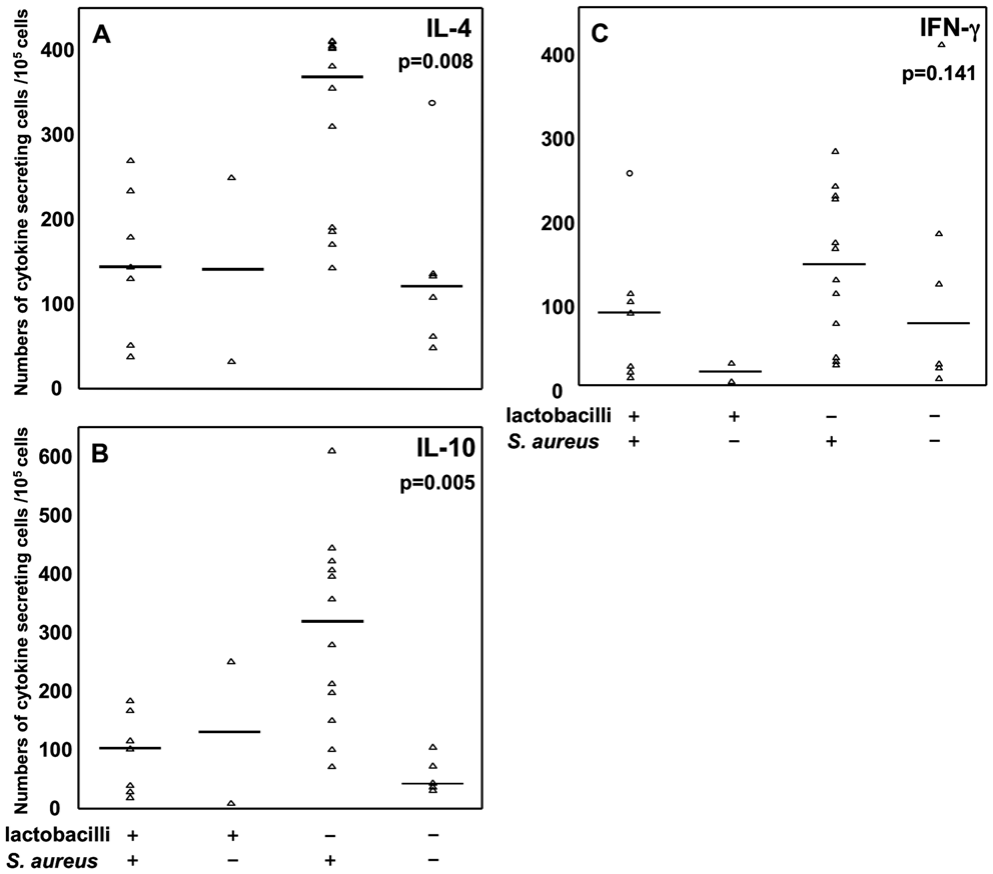
Co-colonization of lactobacilli and *S. aureus* at 2 weeks and cytokine secreting cells, after *in vitro* PHA stimulation at age two. Infants colonized with both *S. aureus* and lactobacilli (n = 7), none (n = 6), only *S. aureus* (n = 12) or only lactobacilli (n = 2) at 2 weeks of age in relation to (A) IL-4−, (B) IL-10−, and (C) IFN-γ producing cells. Triangles represent each individual and lines represent median value.

**Figure 4:**
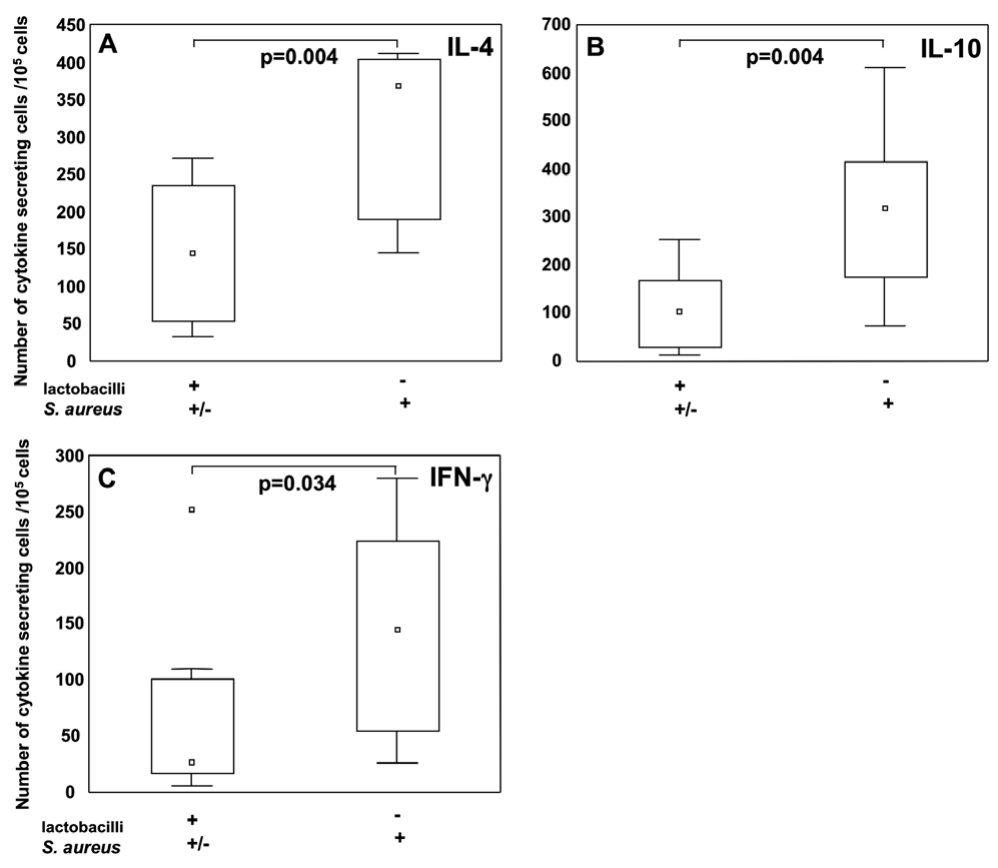
Lactobacilli and *S. aureus* co-colonization at 2 weeks of age in relation to cytokine secreting cells, after *in vitro* PHA stimulation at age two. Infants colonized with only *S. aureus* (n = 12) or infants colonized with lactobacilli (+/− *S. aureus*) (n = 9) with at 2 weeks of age in relation to (A) IL-4−, (B) IL-10−, and (C) IFN-γ-producing cells after PHA stimulation. Boxes cover 25^th^ to 75^th^ percentile and the central square being the median value. Whiskers extend to non-outlier maximum and minimum and squares represents outliers.

As early-life colonization with lactobacilli and *S. aureus* were associated with opposite pattern of cytokine-secreting cells at age two, we investigated early co-colonization with both, none or either of lactobacilli and *S. aureus* in relation to number of cytokine-secreting cells at age two after PHA stimulation. Infants were grouped according to their two-week colonization with lactobacilli and *S. aureus*, being colonized with both, one or none of the bacterial species (Fig. 3). From this grouping it was apparent that colonization with *S. aureus* in the absence of lactobacilli was associated with significantly elevated numbers of cytokine-secreting cells in comparison to the other colonization groups. Therefore, the children were re-grouped based on lactobacilli colonization at two weeks: infants colonized with *S. aureus* but not with lactobacilli (*S. aureus*+ and lactobacilli−) and infants colonized with lactobacilli regardless of *S.aureus* colonization (lactobacilli+ and *S.aureus* +/−). The presence of *S. aureus* in the absence of lactobacilli associated with significantly more IL-4−, IL-10− and IFN-γ producing cells after PHA stimulation at two years of age (Fig. 4A–C). Similar trends were observed when analyzing colonization at one and two months of age for both IL-4 and IL-10 (data not shown).

### Lactobacilli inhibits S. aureus induced T helper cell IFN-γ production in vitro

**Figure 5:**
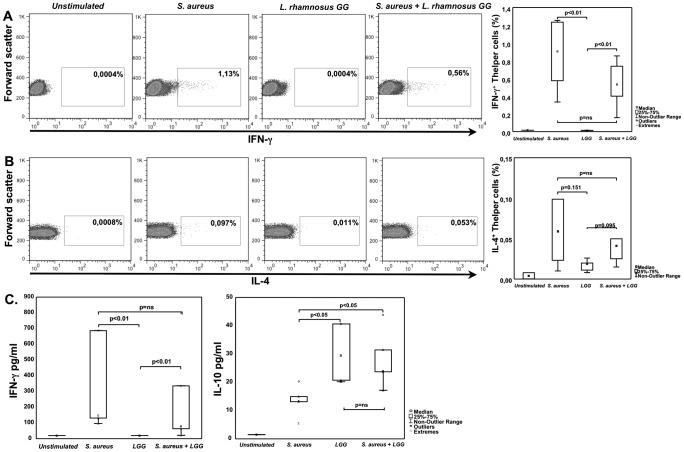
*S. aureus* supernatant induces IFN-γ producing T helper cells and soluble IFN-γ after *in vitro* stimulation of PBMCs. *In vitro* stimulation of PBMCs with *S. aureus* 161.2 supernatant induces higher percentages of CD4^+^ T helper cells positive for IFN-γ (A) and tends to induce higher percentages of IL-4^+^ positive CD4^+^ T helper cells (B). IFN-γ and IL-10 released into culture supernatant shown in (C). Data are representative of 5–6 healthy donors.

Given that colonization with *S. aureus* in the absence of lactobacilli was associated with significantly elevated numbers of cytokine-secreting cells, we aimed to further investigate the immunostimulatory capacity of these bacteria *in vitro*. Supernatants from *L. rhamnosus GG* (LGG) and *S. aureus* 161.2 were added to PBMCs and intracellular IL-4− and IFN-γ production was analyzed with FACS. The release of these cytokines as well as IL-10 was measured with ELISA. We found higher frequencies of IFN-γ (p<0.01) and tendency towards increased IL-4 (p = 0.151) producing CD4^+^ T helper cells induced by *S. aureus* 161.2 supernatant than by LGG supernatant. When both supernatants were added simultaneously to the PBMC cultures, the frequencies of IFN-γ− (p<0.01) and IL-4 (p = 0.095) producing T helper cells were increased compared to LGG alone (Fig. 5 A–B). Further, IFN-γ release into culture supernatant was also higher in *S. aureus* 161.2 stimulated cultures (Fig. 5C). For IL-4, secreted levels were undetectable or extremely low; however detectable only in supernatants from *S. aureus* 161.2 stimulated cells (data not shown). In contrast, IL-10 production was higher in the LGG stimulated cultures as compared to *S. aureus* 161.2 stimulation alone (p<0.05) (Fig. 5C).

## Discussion

Studies of germ free and gnotobiotic mice have uncovered the impact of the microbiota on the maturation of both innate and adaptive immune branches of the system [1]. In humans, the role of the microbiota for immune maturation is not as clear. However, there are reports of associations between microbiota composition and immune-mediated disease, although the underlying mechanisms behind these associations are still largely unknown [9]. Based on the hypothesis that the early-life gut microbiota composition influences infant immune maturation, we have investigated early gut bacterial species in relation to numbers of cytokine-secreting cells at two years of age. We clearly demonstrate that infant gut colonization with certain bacterial species associates with the number of cytokine-secreting cells in a species-specific manner later in childhood. Infant colonization with lactobacilli tended to associate with fewer IL-4−, IL-10− and IFN-γ producing cells at two years of age compared to non-colonized infants after PHA stimulation (Fig. 1A–C). In line with our results, colonization with lactobacilli has previously been reported to associate with lower cytokine responses following allergen stimulation [16]. Also, in a recent paper, intranasally administered lactobacilli to mice resulted in a diminished expression of several pro-inflammatory cytokines, via a TLR-independent pathway [26], suggesting that *Lactobacillus* species generally seem to suppress immune responses.

As for lactobacilli, the early presence of bifidobacteria species has been associated with immune function and allergy development. Although we did not find any consistent associations between early colonization with bifidobacteria and cytokine production at two years of age in this study, early colonization with *Bifidobacterium* species is associated with higher levels of secretory IgA in saliva [15] and reduced allergy prevalence at five years [12], [14].

Gut colonization with the skin/nasal passage bacteria *S. aureus* is common during infancy and probably caused by increased hygienic conditions in the Westernized Countries [27]–[28]. Here, we show that *S. aureus* gut colonization two weeks after birth associates with significantly increased numbers of IL-4− and IL-10 secreting cells, after PHA stimulation at two years of age (Fig. 2A–B). *S. aureus* colonization [11] and exposure to its enterotoxins [25] have been associated with asthma and rhinitis, and also in our study *S. aureus* seems to be more frequently detected early in infants being allergic at the age of five [14].

In children co-colonized with both lactobacilli and *S. aureus* compared to children colonized with *S. aureus* alone, suppressed numbers of IL-4−, IL-10− and IFN-γ secreting cells were found from these children at two years of age (Fig. 3, Fig. 4). This indicates that the simultaneous presence of lactobacilli early in life might modulate an *S. aureus* induced effect on the immune system. Children negative for both species had cytokine-producing cell numbers in the same magnitude as children colonized with lactobacilli, indicating that it is the presence *S. aureus*, and not solely the absence of lactobacilli, that triggers an increased number of cytokine-producing cells. As the majority of infants are colonized with *S. aureus* early in life, we speculate that other species, such as certain *Lactobacillus* spp, might be needed to regulate *S. aureus* triggered responses to avoid an inappropriate immune stimulation. Further, our *in vitro* PBMCs stimulations with *S. aureus 161.2* and LGG support the idea that *S. aureus* induces a cytokine response, which can be suppressed by lactobacilli. The opposing findings regarding IL-10 in relation to *S. aureus* 161.2 may be an *in vitro* and *in vivo* consequence and due to the differences in our experimental set-ups. For the association-study we measured PHA-stimulated T cell cytokine responses, while for the *in vitro* studies we investigated the direct effects of the bacterial species on PBMCs. Thus, other cells, e.g. monocytes, may produce IL-10, which could then explain these contradictory results.

Several potential mechanisms, by which lactobacilli potentially exert their immunosuppressive effects, have been reported. For instance, lactic acid produced by lactobacilli, has been shown to degrade gram-positive bacterial lipoteichoic acid and reduce pathogen-induced cell cytotoxicity [29]. In addition, metabolites from lactic acid producing bacteria have been reported to reduce TLR-induced inflammatory responses [30]. Also, T helper responses, in PBMCs cultures after PHA stimulation, were down regulated, by lactobacilli, in an monocyte-induced IL-10 dependent manner [31] supporting our *in vitro* findings of increased IL-10 levels in LGG stimulated cultures.

Supplementation with different *Lactobacillus* species has been used in allergy prevention, but the results vary between studies [32]–[35]. Still, several probiotic strains have been demonstrated to exert immunomodulatory functions. For example supplementation of *L. gasseri* to allergic school-age children suppressed their PHA-stimulated cytokine responses of IL-12, IL-13 and IFN-γ followed by a reduction of clinical symptom scores [36], which is interesting in relation to the findings presented in our study.

We should acknowledge the relatively low number of individuals in this study, and the findings need to be confirmed in larger studies. However, we only report results that are consistent at several early time points, thus increasing the probability of true findings. Further, it should be mentioned that results of our *in vitro* studies are based on the effects of bacterial supernatants that may not represent the *in vivo* situation in the intestinal tract.

In conclusion, we demonstrate that the early infant microbiota associates with the numbers of cytokine-secreting cells at two years of age, in a genus- and species specific manner, which we further confirmed by *in vitro* stimulations. As different species and strains have different capacity to alter the cytokine responses later in life, the early-life gut microbiota could modulate the risk of developing inflammatory conditions like allergic disease.
